# Colistin and tobramycin resistance during long- term use of selective decontamination strategies in the intensive care unit: a *post hoc* analysis

**DOI:** 10.1186/s13054-015-0838-4

**Published:** 2015-03-25

**Authors:** Bastiaan HJ Wittekamp, Evelien AN Oostdijk, Anne Marie GA de Smet, Marc JM Bonten

**Affiliations:** Julius Center for Health Sciences and Primary Care, University Medical Center Utrecht, Heidelberglaan 100, 3584 CX Utrecht, The Netherlands; Department of Medical Microbiology, University Medical Center Utrecht, Heidelberglaan 100, 3584 CX Utrecht, The Netherlands; CAPE, Critical Care, Anesthesiology, Peri-operative and Emergency Medicine Research Program, University of Groningen, University Medical Center Groningen, Hanzeplein 1, 9700 RB Groningen, The Netherlands

## Abstract

**Introduction:**

Selective decontamination of the digestive tract (SDD) and selective oropharyngeal decontamination (SOD) have been shown to improve intensive care unit (ICU) patients’ outcomes. The aim of this study was to determine the effects of long-term use of SDD and SOD on colistin and tobramycin resistance among gram-negative bacteria.

**Methods:**

We performed a *post hoc* analysis of two consecutive multicentre cluster-randomised trials with crossover of interventions. SDD and SOD were alternately but continuously used during 7 years in five Dutch ICUs participating in two consecutive cluster-randomised trials. In both trials, to measure colistin and tobramycin resistance among gram-negative bacteria, rectal and respiratory samples were obtained monthly from all patients present in the ICU.

**Results:**

The prevalence of tobramycin resistance in respiratory and rectal samples decreased significantly during long-term use of SOD and SDD. (rectal samples risk ratio (RR) 0.35 (0.23 to 0.53); respiratory samples RR 0.48 (0.32 to 0.73), SDD compared to standard care). Colistin resistance in rectal and respiratory samples did not change (rectal samples RR 0.63 (0.29 to 1.38); respiratory samples RR 1.26 (0.35 to 4.57), SDD compared to standard care).

**Conclusions:**

In this study, in a setting with low antimicrobial resistance rates, the prevalence of resistance against colistin and tobramycin among gram-negative isolates did not increase during a mean of 7 years of SDD or SOD use.

**Electronic supplementary material:**

The online version of this article (doi:10.1186/s13054-015-0838-4) contains supplementary material, which is available to authorized users.

## Introduction

Selective digestive tract decontamination (SDD) and selective oropharyngeal decontamination (SOD) aim to eradicate potential pathogenic microorganisms from the digestive tract to prevent infections in intensive care patients. The most commonly used SDD regimen consists of a non-absorbable antimicrobial mouth paste and gastroenteral suspension containing colistin, tobramycin and amphotericin. In addition, systemic broad-spectrum antibiotics are administered during the first four days in the intensive care unit (ICU). SOD consists of the mouth paste only. Both strategies have been associated with lower mortality, shortened length of stay in hospital and ICU, and less ICU-acquired infections such as bacteremia [1-3]. Routine use of SDD and SOD has remained controversial, mainly because of the fear that (long-term) use will increase antibiotic resistance [4,5]. A recent systematic review and meta-analysis failed to demonstrate such an association, but also concluded that more evidence is needed regarding the long-term effects of SDD/SOD on ICU ecology [5]. We, therefore, measured the prevalence of colistin and tobramycin resistance in five ICUs that have continuously been using SDD or SOD for 6 years or longer.

## Methods

The effects of SOD and SDD were evaluated in a cluster-randomised cross-over study between 2004 and 2006 (study I). Each of 13 participating ICUs used SDD, SOD and standard care (no SDD/SOD), as unit-wide measures for 6 months, with the order of the three periods randomised per ICU. Methodological details and results of the study have been published previously [3]. A second cluster-randomised cross-over study (study II) evaluated the effects of SOD and SDD (without standard care period) when applied as unit-wide interventions during 12 months in 16 Dutch ICUs between 2009 and 2013 [6]. Five ICUs participated in both studies, and continued to use SDD as standard care in the interval between studies (2006 to 2009). These ICUs were contacted to verify that no changes in infection control strategies had taken place for the duration of both studies. For both studies, the need for informed consent was waived by the institutional review board. (See Acknowledgements for full details)

During both studies, monthly point prevalence surveys were performed, in which rectal swabs and throat swabs or endotracheal aspirates (respiratory samples) were obtained from all patients present in the ICU on the day of the survey. This included patients who did not receive SDD or SOD at the time of the point prevalence survey. Where possible endotracheal aspirates were obtained, with throat swabs regarded as the best option in non-intubated patients.

### Microbiology methods

Samples were plated on selective agar, including media containing polymyxin and tobramycin in local microbiology laboratories. Screening for colistin resistance was done using plates containing polymyxin B (5 mg/l) in study period I and polymyxin E (4 mg/l) in study period II. Cultures were analysed semi-quantitatively for growth of gram-negative bacteria. Minimum inhibitory concentration (MIC) values for colistin and tobramycin were determined using automated testing. EUCAST cutoff values were used to determine antibiotic resistance to colistin and tobramycin. Bacteria with susceptibility reported as intermediate (I) or resistant (R) were considered resistant. Species with intrinsic resistance to colistin, such as *Morganella*, *Citrobacter* and *Serratia* spp. were excluded from the analysis for colistin resistance. Additional information on the characteristics of the two studies, including microbiology methods used can be found in Additional file 1.

### Statistical analysis

Prevalence for colistin and tobramycin resistance were calculated separately per intervention period by dividing the number of patients with one or more resistant isolates per intervention period by the total number of patients included in the surveys of that intervention period. Patients could participate in multiple sequential surveys during one intervention period. The prevalence of antibiotic resistance is given as percentage with 95% confidence intervals (CI). Relative risks (RR) and 95% CI were calculated to compare the prevalence of resistance between the two study periods.

## Results

**Figure 1:**
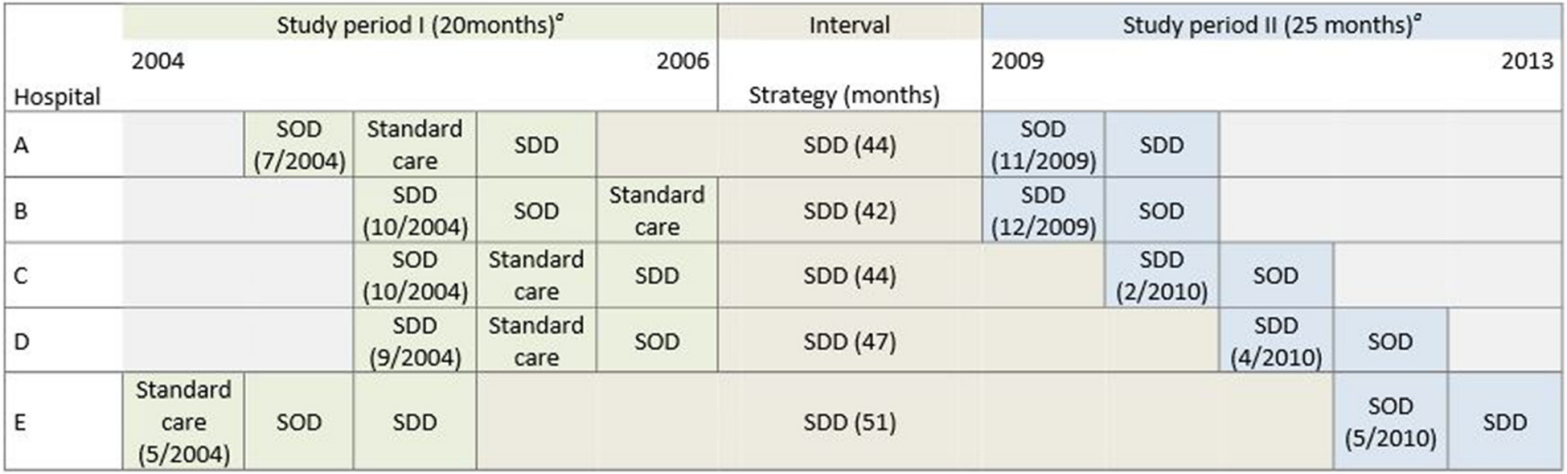
**Time line of the two consecutive studies and interval period. (a)** In both studies interventions were separated by a one month wash-in wash-out period (not shown) [3,6]. The baseline period is the control period in which ICUs used standard care, not including SDD or SOD. All centres continued SDD in the interval period (grey). The end of the intervention period marks the end of the study. ICU, intensive care unit; SDD, selective digestive tract decontamination; SOD, selective oropharyngeal decontamination.

The average duration of SDD/SOD use per ICU was 7.05 years (range 6.8 to 7.5 years), excluding the 6-month standard care period of study period I. A timeline of the two studies can be found in Figure 1. During study period I, 1,007 respiratory and 1,093 rectal samples were obtained from 1,189 patients in the five participating ICUs. During study period II, 1,755 respiratory and 1,808 rectal samples were obtained from 1,865 patients.

**Figure 2:**
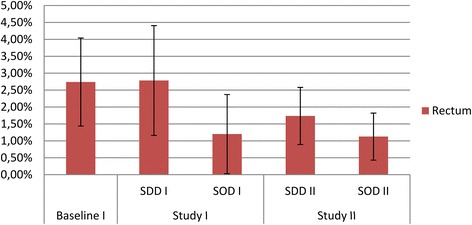
**Colistin resistance in rectal samples.** Prevalence of gram-negative bacteria with intermediate susceptibility (I) or resistant (R) to colistin in rectal samples obtained during study period 1 and 2 respectively.

**Figure 3:**
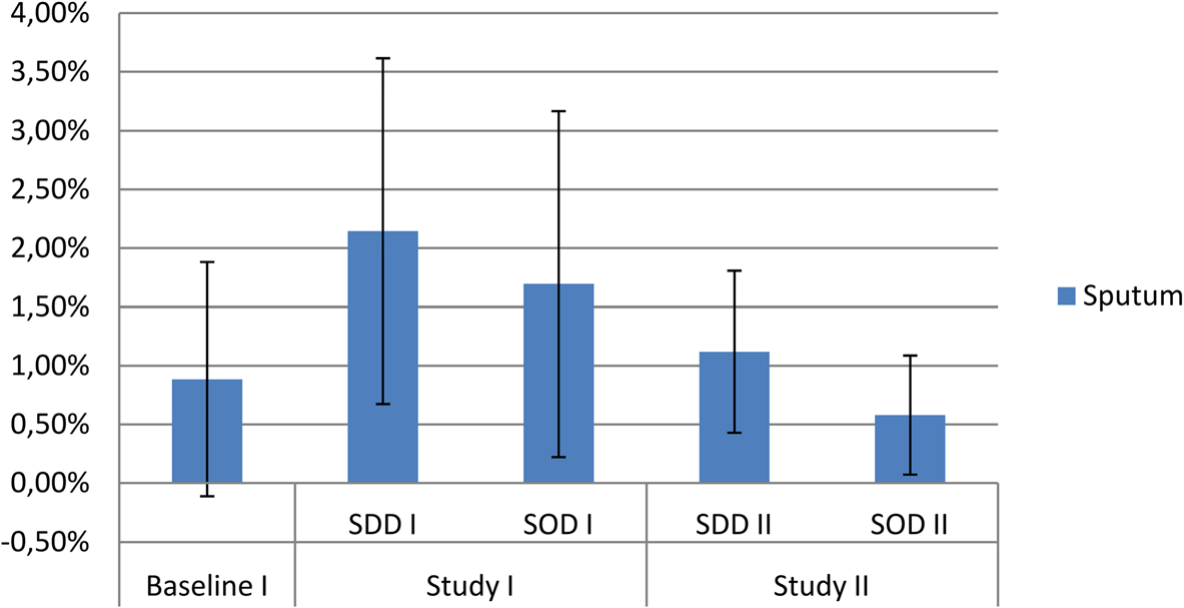
**Colistin resistance in respiratory samples.** Prevalence of gram-negative bacteria with intermediate susceptibility (I) or resistant (R) to colistin in respiratory samples obtained during study period 1 and 2 respectively.

The prevalence for colistin resistance in rectal samples ranged from 1.2% (during SOD) to 2.8% (during SDD) in study period I, and were 1.7% and 1.1% during SDD and SOD, respectively in study period II (Figure 2)*.* In respiratory tract samples, the prevalence for colistin resistance ranged from 0.88% (standard care) to 2.1% (SDD) in study period I and were 1.1% and 0.6% during SDD and SOD in study period II (Figure 3). There were no statistically significant differences between study periods or between intervention periods, except for a significant decrease in colistin resistance in rectal samples during the SOD period of study period II compared to the standard care period of study period I (RR 0.41 (0.17 to 0.98)) (Table 1).

**Table 1:** **Relative risk of colistin and tobramycin resistance**

	***Relative risk (95% CI)***			
	**SDD study II**	**SDD study II**	**SOD study II**	**SOD study II**
	**vs. standard care**	**vs. study I**	**vs. standard care**	**vs. study I**
Colistin				
Rectum	0.63 (0.29-1.38)	0.62 (0.29-1.33)	0.41 (0.17-0.98)	0.94 (0.30-2.97)
Respiratory tract	1.26 (0.35-4.57)	0.52 (0.21-1.31)	0.66 (0.16-2.73)	0.34 (0.10-1.18)
Tobramycin				
Rectum	0.35 (0.23-0.53)	0.64 (0.40-1.04)	0.66 (0.47-0.95)	0.56 (0.39-0.78)
Respiratory tract	0.48 (0.32-0.73)	0.78 (0.49-1.25)	0.42 (0.27-0.64)	0.48 (0.30-0.76)

CI, confidence interval; SDD, selective digestive tract decontamination; SOD, selective oropharyngeal decontamination.

**Figure 4:**
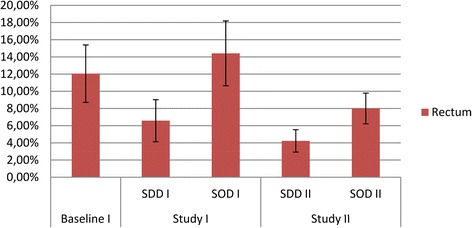
**Tobramycin resistance in rectal samples.** Prevalence of gram-negative bacteria with intermediate susceptibility (I) or resistant (R) to tobramycin in rectal samples obtained during study period 1 and 2 respectively.

**Figure 5:**
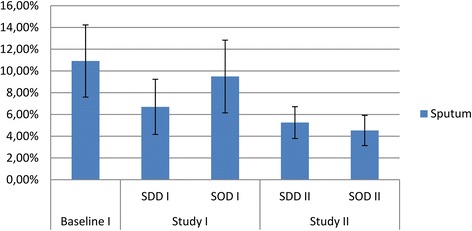
**Tobramycin resistance in respiratory samples.** Prevalence of gram-negative bacteria with intermediate susceptibility (I) or resistant (R) to tobramycin in respiratory samples obtained during study period 1 and 2 respectively.

The prevalence of tobramycin resistance in rectal samples in study period I was lowest during SDD (6.6%), as compared to standard care (RR 0.54 (0.34 to 0.87) and SOD (RR 0.46 (0.29 to 0.72)) (Figure 4). In study period II, the prevalence was 4.2% during SDD (RR 0.64 (0.40 to 1.04) as compared to SDD in study period I) and 8% during SOD (RR 0.56 (0.39 to 0.78) as compared to SOD in study period I) (Table 1). The prevalence for tobramycin resistance in respiratory samples during SDD in study period I was (6.7%), which was lower than during standard care and SOD (RR 0.61 (0.38 to 1.00) and 0.71 (0.42 to 1.18), respectively). In study period II, the prevalence was 5.3% during SDD (RR 0.78 (0.49 to 1.25) as compared to SDD in study period I) and 4.5% during SOD (RR 0.48 (0.30 to 0.76) as compared to SOD in study period I) (Table 1 and Figure 5).

As compared to the standard care period in study period I, the average point prevalence of tobramycin resistance in rectum samples had declined in study period II; from 12.1% in the standard care period of study period I to 4.2% during SDD and 8% during SOD in rectal swabs (RR 0.35 (0.23 to 0.53) and 0.66 (0.47 to 0.95), respectively), and from 10.9% in the standard care period of study period I to 5.3% during SDD (RR 0.48 (0.32 to 0.73) and 4.5% during SOD in respiratory tract samples (RR 0.42 (0.27 to 0.64)) (Table 1).

The identified species and their counts in each study period are available in Additional file 2.

## Discussion

In this longitudinal ecological study, spanning a period of 7 years, we found no evidence of increasing resistance to colistin in ICUs using SDD and SOD. Moreover, resistance to tobramycin among gram-negative bacteria was lower after several years of SDD and SOD.

These findings provide further evidence on the ecological effects of SDD and SOD in settings with low levels of antibiotic resistance, which was previously documented during short-term use [3,6].

The current results support previous findings obtained from two longitudinal studies in Germany and Spain using clinical culture results and surveillance cultures, respectively [7,8]. In a French retrospective study, spanning 6 years, carriage of antibiotic-resistant bacteria based on clinical culture results was compared for individual patients receiving or not receiving SDD, yielding no changes in resistance among gram-negative bacteria [9]. In 17 Dutch ICUs that continuously used SDD or SOD during 4 years of follow-up, there was no increase in resistance against colistin and tobramycin among gram-negative bacteria, while resistance against third-generation cephalosporins and ciprofloxacin decreased during the follow-up period [10].

The current results are based on point prevalence samples obtained from all patients present in the ICU on a predefined moment, also including patients not directly exposed to SDD or SOD, thus reflecting the ICU ecology. If SDD or SOD would directly cause antibiotic resistance in exposed patients, inclusion of non-exposed patients would dilute this effect, creating a bias towards null. However, in a previous analysis of patients receiving SDD or SOD during study period I antibiotic resistance was lower than during standard care [11].

Results of the second cluster-randomised cross-over study, performed in 16 ICUs yielded a 7% and 4% monthly increase in the prevalence of aminoglycoside-resistant gram-negative bacteria in rectal samples during 12 months of SDD and 12 months of SOD, respectively [6]. In addition, SDD was associated with an increase in aminoglycoside resistance genes in the non-culturable intestinal flora in some patients [12]. These findings are in contrast with the current findings, which might be related to differences in the duration of follow-up (7 years versus 24 months), study population (five ICUs versus 16 ICUs), and detection methods (conventional microbiology versus metagenomics approaches). Careful monitoring of aminoglycoside resistance should, therefore, be performed during SDD or SOD.

Clonal spread of colistin-resistant extended-spectrum beta-lactamase (ESBL)-producing *Klebsiella pneumonia* has been described after introduction of SDD during an outbreak that could not be controlled with classical infection control measures [13]. That situation markedly differed from the non-outbreak study settings. In the Netherlands, the prevalence of multidrug resistance among both gram-negative and gram-positive bacteria in general is low [6,10,11].

This study has some limitations. Adjustment for secular trends of antibiotic resistance was not possible, since there was no data from ICUs that did not use SDD or SOD. Yet, in an analysis of trends of resistance against aminoglycosides and colistin among enterobacteriaceae between 2008 and 2012 in 13 Dutch hospitals in which no SDD or SOD was used a significant change could not be demonstrated [10].

Furthermore, distributions of MIC values were lacking in the current study.

Changes in case mix on the ICUs and implementation of other interventions (that is infection control measures) that influence the prevalence of antibiotic resistance could have occurred during the interval between the two studies, although based on reports of the participating hospitals we have no indication that either of these took place.

Analysis of third-generation cephalosporin resistance was not performed as different selective culture media were used for screening in both study periods. Moreover, polymyxin B (5 mg/l) and polymyxin E (4 mg/l) were used for screening in study periods I and II, respectively, but it is unlikely that this had consequences for our findings, since there is complete cross-resistance between colistin and polymyxin B [13].

## Conclusions

This study did not find an increase in the prevalence of resistance against colistin and tobramycin among gram-negative isolates during a mean of 7 years of SDD or SOD use.

The effect of SDD and SOD in settings with higher levels of antibiotic resistance than the Netherlands remains to be determined.

## Key message

- The continued use of SDD or SOD for 7 years in a setting with low levels of antibiotic resistance did not lead to an increase in the (point) prevalence of colistin and tobramycin resistance among gram-negative bacteria.

## Additional files

Additional file 1: Table S2. Characteristics of the methods and interventions in the two studies. Additional file 2: Table S3. Count of resistant isolates of a species during each study period.

## Abbreviations

CI: confidence interval
ESBL: extended-spectrum beta-lactamase
ICU: intensive care unit
MIC: minimum inhibitory concentration
RR: relative risk
SDD: selective digestive tract decontamination
SOD: selective oropharyngeal decontamination
spp: species
