# Supplementary material for: Colistin and tobramycin resistance during long- term use of selective decontamination strategies in the intensive care unit: a post hoc analysis
Source: Crit Care. 2015 Mar 25;19(1):113. doi: 10.1186/s13054-015-0838-4 (PMC4373110; doi:10.1186/s13054-015-0838-4)
Supplement: Additional file 1: Table S2. — Characteristics of the methods and interventions in the two studies. [file 13054_2015_838_MOESM1_ESM.pdf]

Table 2: Characteristics of the two studies

	Study period I (3)	Study period II (6)
Years in which inclusion took place	2004-2006	2009-2013
Participating hospitals (n)	13	16
Patients included (n)	5939	11997
Duration of intervention		
SDD	6 months	12 months
SOD	6 months	12 months
Standard care	6 months	*NA*
Inclusion criteria to receive SDD/SOD	Mechanical ventilation > 48 hours and/or Length of stay > 72 hours	Mechanical ventilation > 48 hours
Antibiotics used in SDD/SOD		
Tobramycin	Mouthpaste: 2% concentration<br>Suspension: 80mg[a]	*idem*
Colistin	Mouthpaste: 2% concentration<br>Suspension: 100mg[a]	*idem*
Amphotericin	Mouthpaste: 2% concentration<br>Suspension: 500mg[a]	*idem*
Systemic prophylaxis	Cefotaxim 4000mg daily[a]	*idem*
**Methods**		
Point prevalence surveys available (n)	18	24
Culture media used for screening in point prevalence surveys	McConkey with<br>- polymyxin B 50iu/ml (5mg/l)<br>- tobramycin 8mg/l<br>- cefotaxim 8mg/l<br>- ciprofloxacin 2mg/l	McConkey with<br>- polymyxin E (colistin) 4mg/l<br>- tobramycin 8mg/l<br>ESBL chromogenic agar<br>VRE chromogenic agar

[a] only during SDD

Abbreviations: SDD: selective digestive tract decontamination; SOD: selective oropharyngeal decontamination; NA: not applicable; ESBL: extended spectrum beta-lactamase; VRE vancomycin resistant enterococcus
